# IL-12p40 deletion aggravates lipopolysaccharide-induced cardiac dysfunction in mice

**DOI:** 10.3389/fcvm.2022.950029

**Published:** 2022-09-16

**Authors:** Menglin Liu, Zhen Wang, Jishou Zhang, Di Ye, Menglong Wang, Yao Xu, Mengmeng Zhao, Yongqi Feng, Xiyi Lu, Heng Pan, Wei Pan, Cheng Wei, Dan Tian, Wenqiang Li, Jingjun Lyu, Jing Ye, Jun Wan

**Affiliations:** ^1^Department of Emergency, Renmin Hospital of Wuhan University, Wuhan, China; ^2^Department of Cardiology, Renmin Hospital of Wuhan University, Wuhan, China

**Keywords:** sepsis, IL-12p40 deletion, cardiac dysfunction, monocytes, LPS

## Abstract

**Background:**

Cardiac dysfunction is one of the most common complications of sepsis and is associated with the adverse outcomes and high mortality of sepsis patients. IL-12p40, the common subunit of IL-12 and IL-23, has been shown to be involved in a variety of inflammation-related diseases, such as psoriasis and inflammatory bowel disease. However, the role of IL-12p40 in lipopolysaccharide (LPS)-induced cardiac dysfunction remains obscure. This study aimed to explore the role of IL-12p40 in LPS-induced cardiac dysfunction and its potential mechanisms.

**Methods:**

In this study, mice were treated with LPS and the cardiac expression of IL-12p40 was determined. Then, IL-12p40^–/–^ mice were used to detect the role and mechanisms of IL-12p40 in LPS-induced cardiac injury. In addition, monocytes were adoptively transferred to IL-12p40^–/–^ mice to explore their effects on LPS-induced cardiac dysfunction.

**Results:**

The results showed that cardiac IL-12p40 expression was significantly increased after treated with LPS. In addition, IL-12p40 deletion significantly aggravated LPS-induced cardiac dysfunction, evidenced by the increased serum levels of cardiomyocyte injury markers and heart injury scores, as well as by the deteriorated cardiac function. Moreover, IL-12p40 deletion increased LPS-induced monocyte accumulation and cardiac expression of inflammatory cytokines, as well as enhanced the activation of the NF-κB and MAPK pathways. Furthermore, adoptive transfer WT mouse monocytes to IL-12p40^−/−^ mice alleviated LPS-induced cardiac dysfunction and decreased the phosphorylation of p65.

**Conclusion:**

IL-12p40 deletion significantly aggravated LPS-induced cardiac injury and cardiac dysfunction in mice by regulating the NF-κB and MAPK signaling pathways, and this process was related to monocytes. Therefore, IL-12p40 show a protective role in SIC, and IL-12p40 deficiency or anti-IL-12p40 monoclonal antibodies may be detrimental to patients with SIC.

## Introduction

Sepsis is a systemic inflammatory response syndrome (SIRS) caused by severe infection, surgery, burns, trauma, etc., characterized by an imbalance between inflammatory and anti-inflammatory responses in the body. It can cause septic shock and multiple organ dysfunction syndrome (MODS). Sepsis-induced cardiac dysfunction (SIC) is the most common complication in clinical sepsis and is associated with adverse outcomes and high mortality in sepsis patients (1, 2). It is mainly characterized by ventricular enlargement, myocardial contraction and/or diastolic dysfunction (3). Although numerous studies have paved the way to understand the underlying pathogenesis of SIC (4), the specific mechanism is still not clear. Some studies have reported that the potential mechanism of SIC is a result of the complex interaction of inflammation, oxidative stress, autophagy and apoptosis (4–6) rather than due to a single factor alone.

Previous studies have shown that cytokines participate in the pathological process of sepsis by regulating the immune–inflammatory response of the body. Feng et al. reported that the patients with severe sepsis and septic shock had significantly high serum levels of IL-6 and IL-18 (7). Furthermore, Huan et al. reported that the expression level of IL-35 was decreased in mouse heart tissue after treatment with LPS (8). Therefore, some researchers have attempted modulate these cytokines to reduce the disadvantages of the sepsis-related host response. Panacek et al. found that the anti-TNF-α antibody afelimomab can significantly decrease the 28-day mortality rate of sepsis patients whose serum IL-6 level is over 1,000 pg/ml (9). Moreover, the anti-IL-6 receptor (IL-6R) antibody tocilizumab can improve the prognosis of critical COVID-19 patients, as it can bind to IL-6R and block downstream signal transduction (10).

IL-12p40 is the common subunit of IL-12 and IL-23, which are the proinflammatory factors of the IL-12 family. It can be produced by activated inflammatory cells including monocytes, macrophages, dendritic cells (DCs) and neutrophils (11, 12). To date, some studies have explored the roles of IL-12p40 in autoimmune disease (13), inflammatory responses (14), fibrosis (15) and allograft rejection (16). However, in different diseases, the biological effects of IL-12p40 are not fixed and depending on the inflammatory microenvironment (17). Eriksson et al. found that IL-12p40 deletion can protect mice from autoimmune myocarditis (18). Yao et al. reported that IL-12p40 deletion can induces cholangitis and fibrosis in interleukin-2Rα(^−/−^) mice (19). Furthermore, Prando et al. found that patients with IL-12p40 deficiency were susceptibility to mycobacterial disease and salmonellosis disease (20). However, whether IL-12p40 plays a role in sepsis and sepsis-induced cardiac dysfunction is unknown. In this study, we aimed to identify the function of IL-12p40 in sepsis-induced cardiac dysfunction and to explore its underlying mechanisms.

## Materials and methods

### Animals and experimental model

IL-12p40 knockout (IL-12p40^−/−^) mice with a C57BL/6J background were purchased from the Institute of Model Zoology, Nanjing University (imported from the Jackson Laboratory), and wild-type (WT) mice in the same brood were used as controls (21, 22). All mice were housed in the specific-pathogen-free mouse room of Renmin Hospital of Wuhan University, in which the temperature (20–22°C) and humidity (50 ± 5%) were relatively constant, and the mice could freely obtain water and food. The study was approved by the Animal Care and Use Committee of Renmin Hospital of Wuhan University, and the Care and Use of Laboratory Animals were performed in accordance with the NIH Guidelines revised in 2011.

In the first experiment, male WT mice aged 8–10 weeks were randomly divided into a Saline group (*n* = 10) and an LPS group (*n* = 30). Mice in the LPS group were intraperitoneally injected with 10 mg/kg LPS (055:B5, Sigma-Aldrich, USA) (23), while those in the Saline group were intraperitoneally injected with an isovolumetric dose of saline. Every 10 mice in the LPS group were sacrificed with isoflurane at 3, 6, and 12 h after treatment with LPS. Mice in the Saline group were sacrificed in the same way at 6 h after treatment with saline. The heart tissues of all the mice were harvested, and cardiac IL-12p40 expression levels were detected by qRT–PCR and western blotting. In the second experiment, 8- to 10-week-old WT mice and IL-12p40^−/−^ mice were selected and randomly divided into Saline + WT group (*n* = 10), Saline + KO group (*n* = 10), LPS + WT group (*n* = 10) and LPS + KO group (*n* = 10). Mice in the LPS + WT and LPS + KO groups were given LPS at a dose of 10 mg/kg, while those in the other two groups were given an isovolumetric dose of saline. Six hours later, all the mice were anesthetized and underwent cardiac ultrasound. After that, all of them were sacrificed, and serum and heart tissue samples were obtained for further measurement. In the third experiment, 8- to 10-week-old IL-12p40^−/−^ mice were subjected to the adoptive transfer of WT monocytes or IL-12p40^−/−^ monocytes (10^6^ cells/mouse) from the tail vein (24). On the second day, all IL-12p40^−/−^ mice that had received WT monocytes or IL-12p40^−/−^ monocytes were divided into KO+WT Mono group (*n* = 6), KO+WT Mono+LPS group (*n* = 6), KO+KO Mono group (*n* = 6) and KO+KO Mono+LPS group (*n* = 6). Then, mice in the KO+WT Mono+LPS and KO+KO Mono+LPS groups received 10 mg/kg LPS, while those in the other two groups received an isovolumetric dose of saline. Six hours later, all the mice were anesthetized and underwent cardiac ultrasound. Then, all of them were sacrificed, and serum and heart tissue samples were obtained for further measurement.

### Echocardiography

The cardiac function of mice was evaluated by echocardiography as described in our previous study (25). In brief, transthoracic echocardiography was performed using a Mylab30CV ultrasound (Biosound Esaote), and data on left ventricular end-diastolic diameter (LVEDd), left ventricular end-systolic diameter (LVESd), left ventricular end-diastolic volume (LVEDd), left ventricular end-systolic volume (LVESd), ejection fraction (LVEF) and fractional shortening (LVFS) were obtained for 10–15 cardiac cycles.

### Biochemical determination

The creatine kinase-myocardial band (CK-MB) and lactate dehydrogenase (LDH) were assessed as indices of cardiomyocyte injury. Serum concentrations of CK-MB and LDH were detected according to the manufacturer's instructions, and all kits were purchased from Nanjing Jiancheng Bioengineering Institute, China.

### Quantitative real-time PCR

Total RNA was extracted from heart tissues using TRIzol reagent and reverse transcribed to cDNA according to a previous protocol (26). Subsequently, quantitative real-time PCR (qRT–PCR) was performed using a LightCycler 480 (Roche, Switzerland) according to the manufacturer's recommendation. The expression of glyceraldehyde-3-phosphate dehydrogenase (GAPDH) was quantified as an internal control. All the primer sequences used in our study are shown in Table 1.

**Table 1:** All the primer sequences in this study.

**Gene**	**Forward primer (5^′^-3^′^)**	**Reverse primer (5^′^-3^′^)**
ANP	ACCTGCTAGACCACCTGGAG	CCTTGGCTTATCTTCGGTACCGG
BNP	GAGGTCACTCCTATCCTCTGG	GCCATTTCCTCCGACTTTTCTC
IL-1β	GGGCCTCAAAGGAAAGAATC	TACCAGTTGGGGAACTCTGC
IL-6	AGTTGCCTTCTTGGGACTGA	TCCACGATTTCCCAGAGAAC
IL-17	TCCAGAAGGCCCTCAGACTA	AGCATCTTCTCGACCCTGAA
TNF-α	CCCAGGGACCTCTCTCTAATC	ATGGGCTACAGGCTTGTCACT
INF-γ	ACTGGCAAAAGGATGGTGAC	TGAGCTCATTGAATGCTTGG
GAPDH	ACTCCACTCACGGCAAATTC	TCTCCATGGTGGTGAAGACA

### Western blot analysis

The extraction of protein from heart tissues and western blotting were performed according to methods described previously (27). In brief, the heart tissues were lysed by RIPA buffer and ultrasound successively. Total protein was collected from each heart sample and quantified with a BCA Protein Assay Kit (Thermo Fisher Scientific). Then, the proteins (50 μg per sample) were separated by SDS-PAGE and transferred to PVDF membranes (Millipore, Beijing, China). The PVDF membranes were then blocked for 1.5 h with specific 5% non-fat dried milk and incubated with primary antibodies overnight at 4°C. The primary antibodies included anti-IL-12p40, anti-Bcl-2, anti-Bax, anti-c-caspase3, anti-STAT1, anti-p-STAT1, anti-p-p65, anti-p65, anti-CD14, anti-CD16, anti-ERK, anti-p-ERK, anti-p38, anti-p-p38, anti-JNK, anti-p-JNK and anti-GAPDH. Finally, the membranes were incubated with secondary antibodies and scanned with an Odyssey infrared imaging system (LI-COR, USA). The protein expression level of GAPDH was used as an internal control to analyse the expression levels of the target proteins.

### Histological analysis

Hearts were arrested in 10% KCl solution immediately after being obtained. After fixation with 10% formalin for 48 h, the heart specimens were embedded in paraffin and then sliced into 5-μm sections. The heart injury score and myocardial collagen volume were analyzed by hematoxylin and eosin (HE) staining and masson's trichrome staining, respectively. Moreover, these sections were also subjected to immunofluorescence staining. In brief, the sections were incubated with primary antibodies against CD14 (R&D Systems, USA), against CD16 (R&D Systems, USA) and against p-p65 (Abcam, United Kingdom) overnight at 4°C. Then, the sections were incubated with secondary antibodies [anti-rabbit HRP reagent (Gene Tech, Shanghai, China)] and 40,6-diamidino-2-phenylindole [DAPI (Gene Tech, Shanghai, China)]. All the figures were captured with fluorescence microscope, and Image Pro Plus 6.0 (Media Cybernetics, Bethesda, MD, United States) was used for relative quantification.

### TdT-mediated dUTP nick-end labeling (TUNEL) assay

The heart specimens described above were deparaffinized with toluene and dehydrated with ethanol according to the procedures previously described in our previous study (28). Then, a TUNEL kit (Millipore, United States) was used to assess the apoptosis of myocardial tissue.

### Flow cytometry

Flow cytometry of mouse spleen tissue was performed as described previously (29). In brief, isolated cell suspensions from spleens were filtered, centrifuged, resuspended and blocked with a CD16/32 antibody. Then, the cell suspensions were stained with primary antibodies for 30 min at 4°C in the dark. Flow cytometry analysis was performed on a BD FACS Calibur flow cytometer (BD Biosciences, San Jose, CA, USA).

### Monocyte transduction

Monocytes were obtained according to previous reports (30, 31). Briefly, tibias and femurs were collected from WT and IL-12p40^−/−^ mice. Serum-free α-MEM was injected with a syringe into the bone marrow cavities of tibias and femurs to flush out the cells in the cavities. This process was repeated several times until all the cells in the marrow cavities were flushed out. All bone marrow cell suspensions from IL-12p40^−/−^ mice were collected in a petri dish, while those from WT mice were collected in a separate petri dish. After filtration and centrifugation, bone marrow cell suspensions were lysed with sterile cell lysate. Then, the bone marrow cell suspensions were mixed with FBS, PS and α-MEM and seeded in each well of 6-well culture plates. After overnight incubation in a humid incubator with 5% CO2, the supernatants were discarded from the 6-well culture plates, and the adherent cells were treated for 3 days with monocyte colony-stimulating factor (MC-SF), FBS, PS and α-MEM. All cytokines were purchased from R&D Systems. Monocytes were then cultured in fresh serum-free α-MEM medium at a density of 5 × 10^6^ cells/ml. These monocytes from WT or IL-12p40^−/−^ mice were transferred into each IL-12p40^−/−^ recipient mouse through the tail vein at a dose of 10^6^ cells per mouse (24).

### Statistics

All the results are presented as the mean ± SD. One-way analysis of variance (ANOVA) or multiactor analysis of variance was used for comparison of the mean between the groups. A *p* < 0.05 was considered significant.

## Results

### LPS treatment increases cardiac IL-12p40 expression in mice

**Figure 1:**
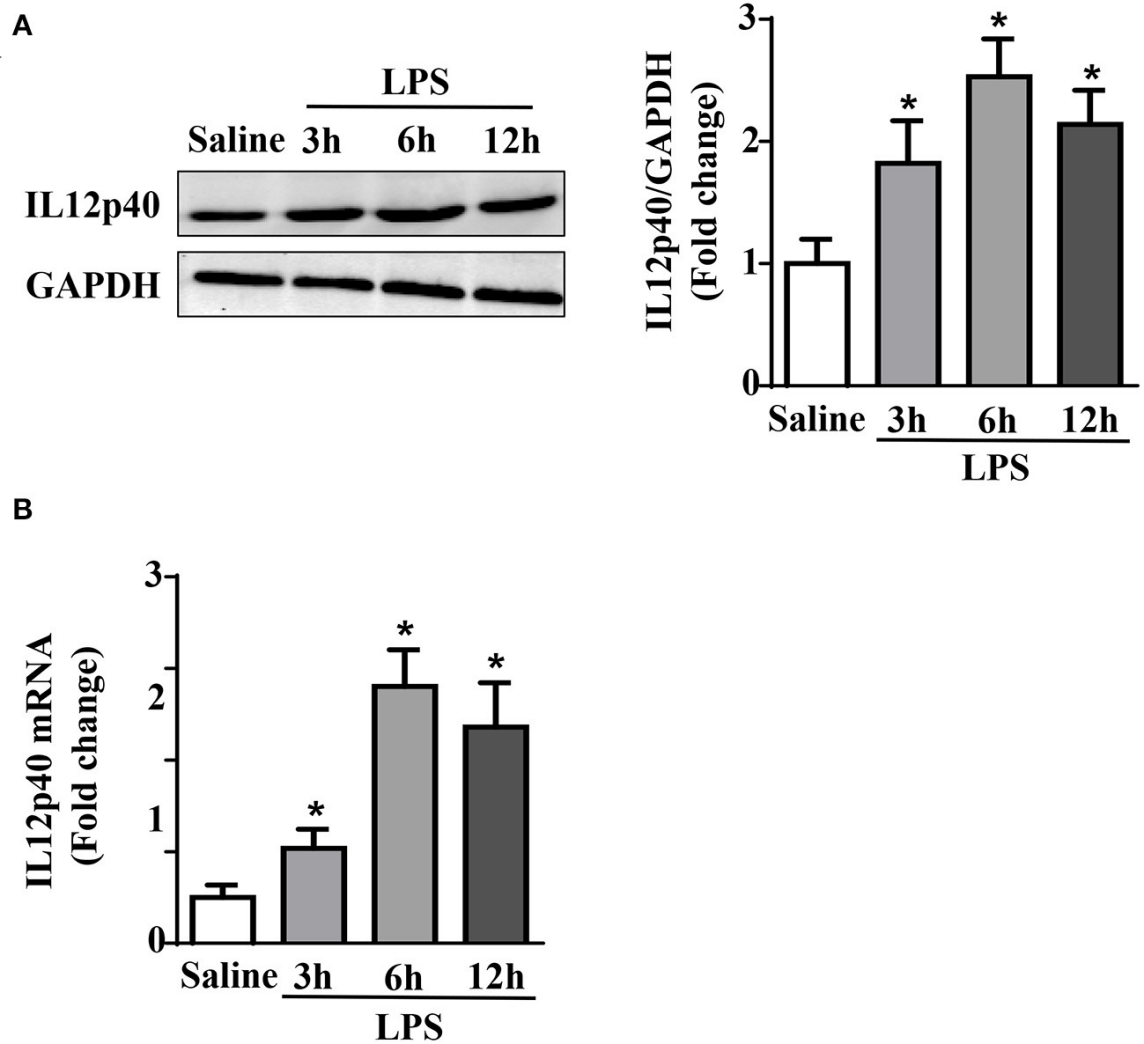
LPS treatment increases cardiac IL-12p40 expression in mice. **(A)** Western blot analysis of IL-12p40 protein levels in the hearts of each group (*n* = 5). **(B)** qRT-PCR analysis of IL-12p40 mRNA expression levels in the hearts of each group (*n* = 6). *P < 0.05 compared with the Saline group.

The results of qRT-PCR and Western blot showed that within 6 h after treated with LPS, the mRNA and protein levels of IL-12p40 in the mouse myocardium increased gradually. However, after 12 h, both the mRNA and protein levels of IL-12p40 showed downwards trends. Moreover, the mRNA and protein levels of IL-12p40 in mice treated with LPS at 3, 6, and 12 h were significantly different from those in mice in the Saline group (Figure 1).

### IL-12p40 deletion aggravates LPS-induced cardiac injury and cardiac dysfunction in mice

**Figure 2:**
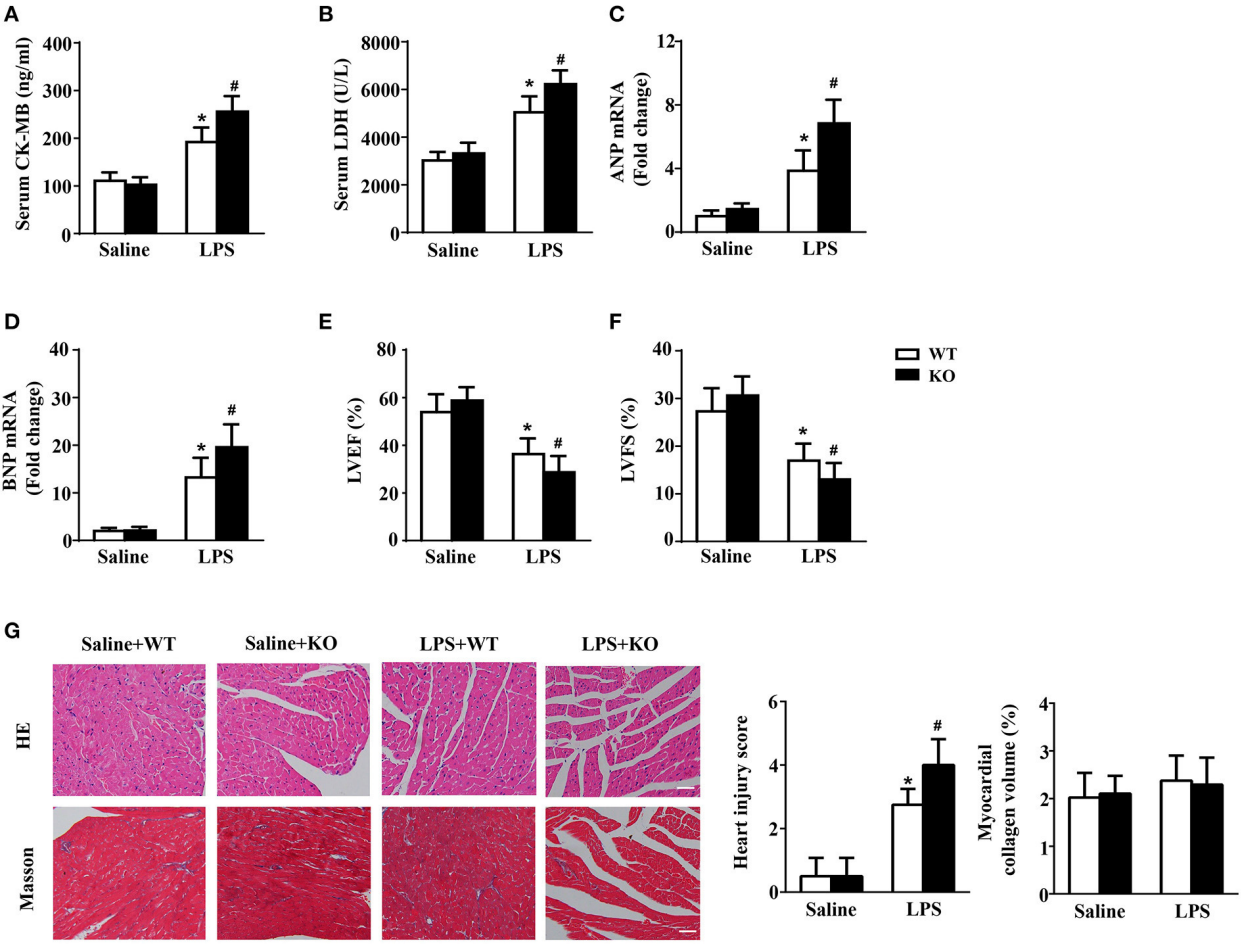
IL-12p40 deletion aggravates LPS-induced cardiac injury and cardiac dysfunction in mice. **(A,B)** The levels of LDH and CK-MB in serum of mice in each group (*n* = 6). **(C,D)** qRT-PCR analysis of ANP and BNP mRNA expression levels in the hearts of mice in each group (*n* = 6). **(E,F)** Echocardiography analysis of LVEF and LVFS of mice in each group (*n* = 6). **(G)** HE and masson's trichrome stainings and the quantitative results of heart tissues in each group (*n* =6; scale bar, 100 μm). *P < 0.05 compared with the Saline group. ^#^*P* < 0.05 compared with the LPS+WT group.

The results of biochemical determination showed that treatment with LPS dramatically increased the LDH and CK-MB levels in the serum of mice, and IL-12p40 deletion further increased their levels in mice treated with LPS (Figures 2A,B). The results of qRT-PCR also showed that the levels of ANP and BNP in the cardiac tissue of mice were obviously increased after treatment with LPS and were further increased after IL-12p40 deletion (Figures 2C,D). In addition, the echocardiography results showed that treatment with LPS significantly reduced the LVEF and LVFS of mice; however, IL-12p40 deletion further reduced the LVEF and LVFS of mice (Figures 2E,F). Furthermore, the histological examination revealed that the heart injury scores of mice treated with LPS were significant higher than those of mice treated with saline, and IL-12p40 deletion could further increase the heart injury scores of mice treated with LPS (Figure 2G). However, there was no significant difference in myocardial collagen volume among all the groups (Figure 2G).

### IL-12p40 deletion increases the activation of NF-κB and MAPK signaling pathways and aggravates cardiac inflammation in mice treated with LPS

**Figure 3:**
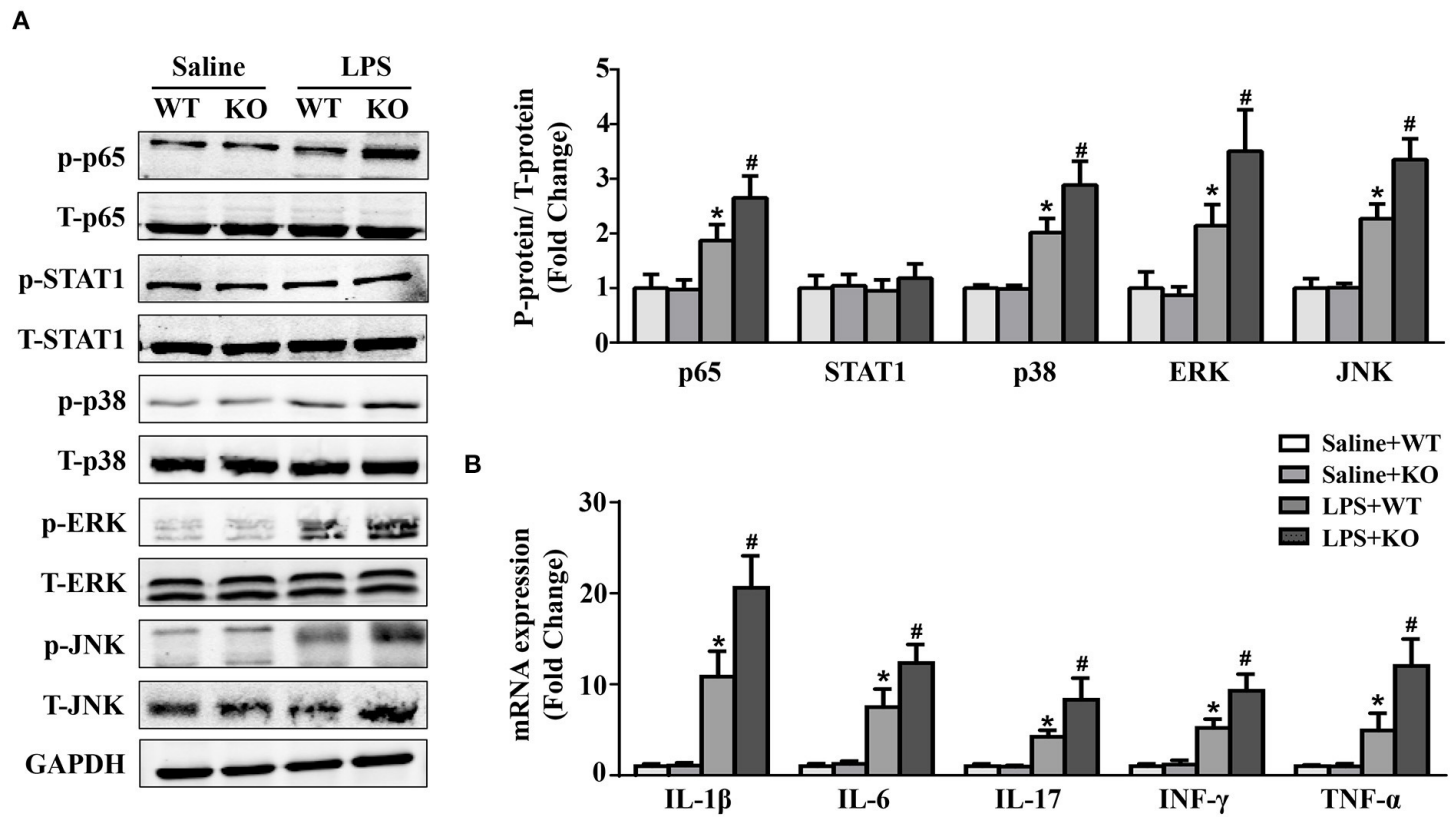
IL-12p40 deletion increases the phosphorylation of NF-κB and MAPK signaling pathways and aggravates cardiac inflammation in mice treated with LPS. **(A)** Western blot analysis of T-ERK, p-ERK, T-p38, p-p38, T-JNK, p-JNK, T-STAT1, p-STAT1, T-P65, and p-P65 protein levels in the hearts of mice and the ratios of p-ERK/T-ERK, p-p38/ T-p38, p-JNK/T-JNK, p-P65/T-P65, and p-STAT1/T-STAT1 in each group (*n* = 5). **(B)** qRT-PCR analysis of IL-1β, IL-6, IL-17, TNF-α and INF-γ mRNA expression levels in the hearts of mice in each group (*n* =6). *P < 0.05 compared with the Saline group. ^#^P < 0.05 compared with the LPS+WT group.

JAK/STAT1 and NF-κB are the signaling pathways of inflammatory responses, while the MAPK signaling pathway is involved in the pathogenesis of LPS-induced cardiac injury. The results showed that LPS stimulation could significantly increase the phosphorylation of p65, p38, ERK and JNK, which were further increased by IL-12p40 deletion (Figure 3A). However, neither LPS stimulation nor IL-12p40 deletion had significant effect on the phosphorylation of STAT1 (Figure 3A). Furthermore, the qRT–PCR results showed that the mRNA levels of the proinflammatory cytokines IL-1β, IL-6, IL-17, TNF-α, and INF-γ were markedly increased after treatment with LPS (Figure 3B). And IL-12p40 deletion could further increase the mRNA levels of these inflammatory cytokines (Figure 3B).

### IL-12p40 deletion increases monocyte infiltration

**Figure 4:**
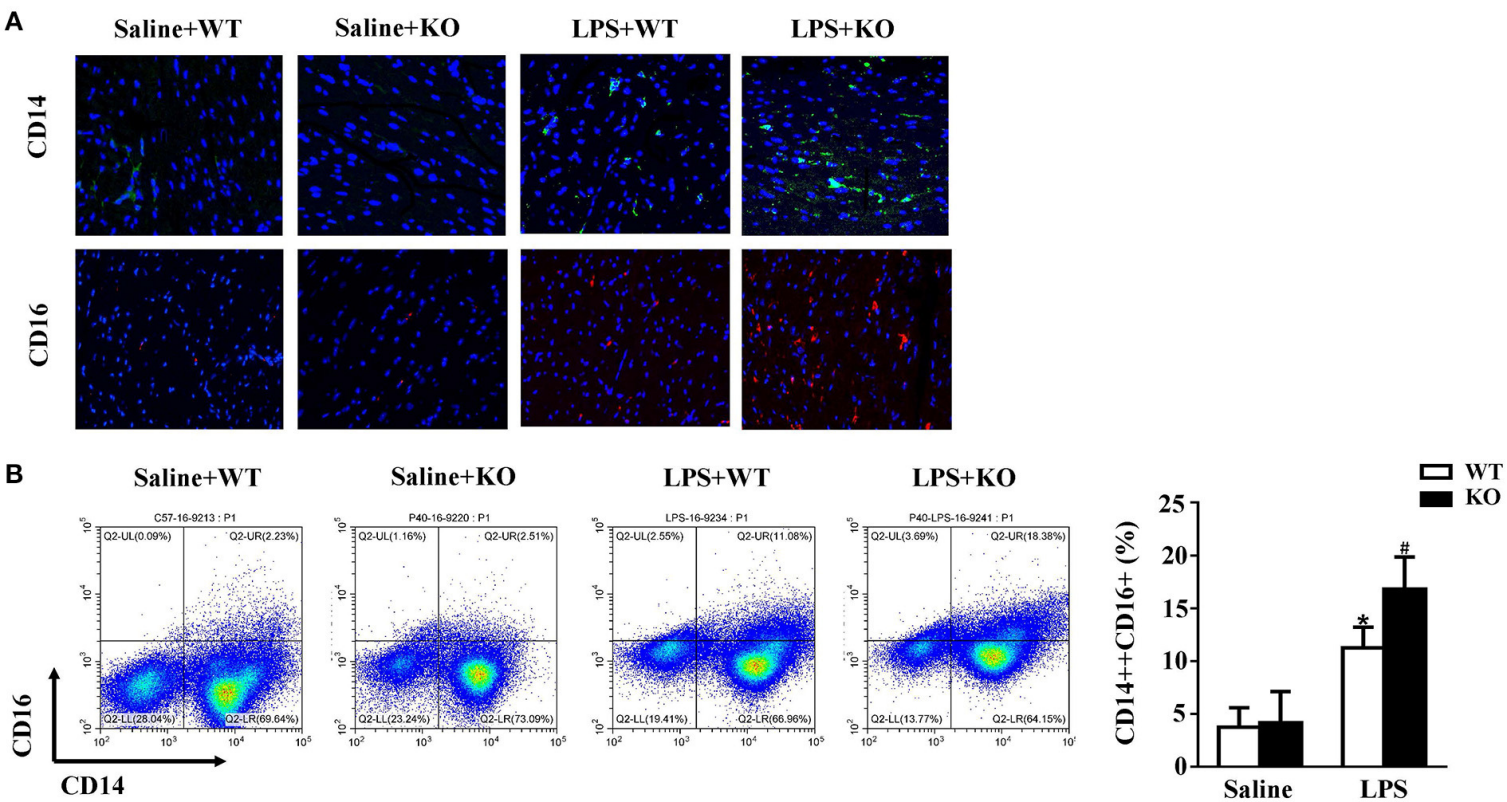
IL-12p40 deletion increases monocytes infiltration. **(A)** The immunofluorescence analysis of CD14 and CD16 in heart sections of each group (*n* = 6; scale bar, 50 μm). **(B)** Flow cytometry analysis of CD14++CD16+monocyte percents in spleen tissues of mice in each group (*n* = 6). *P < 0.05 compared with the Saline group. ^#^*P* < 0.05 compared with the LPS+WT group.

Monocytes are natural inflammatory cells and are involved in the inflammatory response of sepsis (32, 33). Thus, we also evaluated the infiltration of monocytes in mice. CD14 and CD16 are the surface molecules of monocytes. The immunofluorescence results showed that the infiltration of monocytes in the hearts of mice was significantly increased after treatment with LPS. Interestingly, these changes were obviously exacerbated by IL-12p40 deletion (Figure 4A). Furthermore, the results of flow cytometry also showed that LPS stimulation increased the infiltration of monocytes in the spleen, which was further exacerbated by IL-12p40 deletion (Figure 4B).

### IL-12p40 deletion aggravates LPS-induced myocardial apoptosis

**Figure 5:**
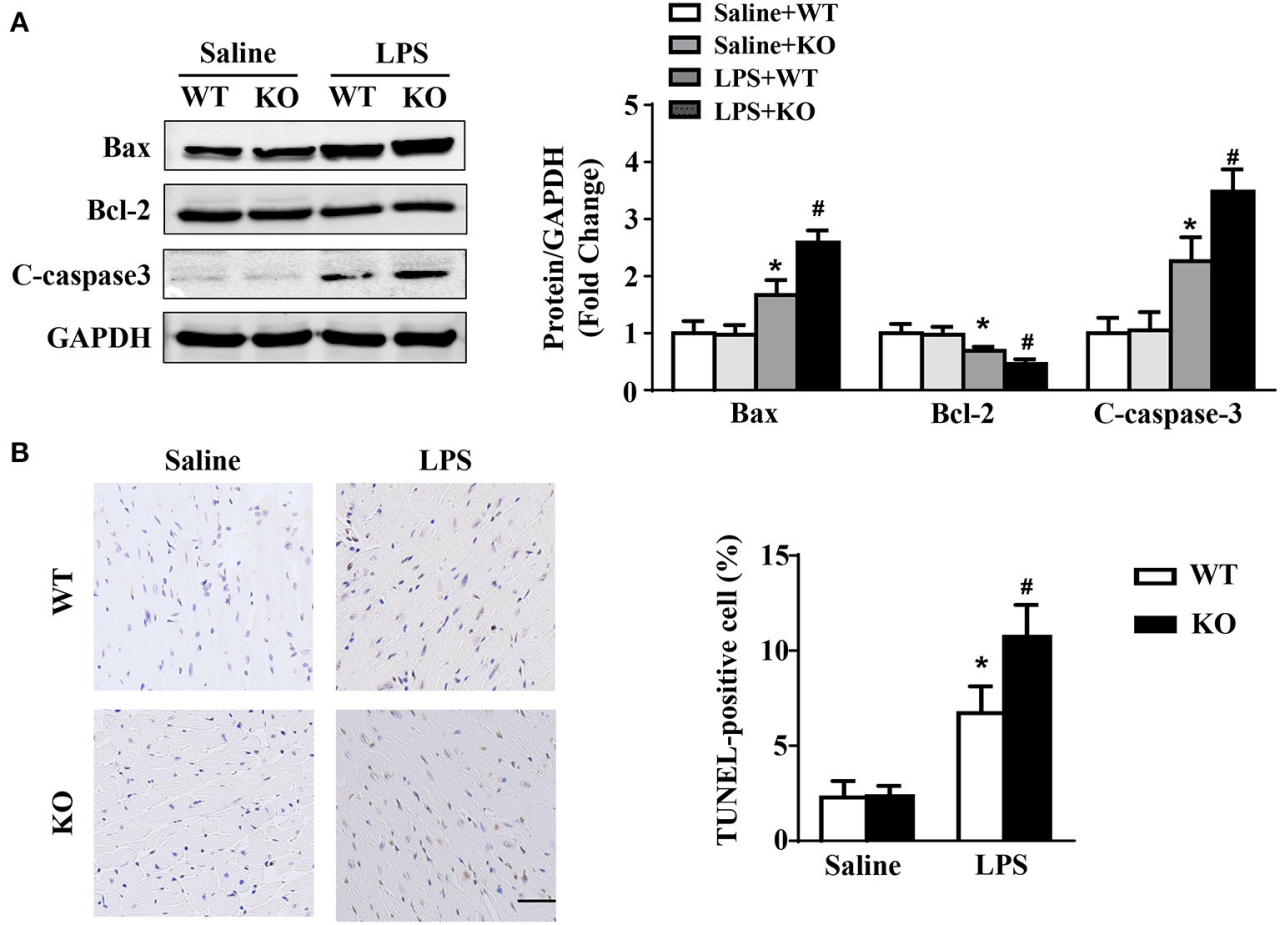
IL-12p40 deletion aggravates LPS-induced myocardial apoptosis. **(A)** Western blot analysis of Bax, Bcl-2 and C-caspase-3 protein levels in heart tissues of each group (*n* = 5). **(B)** TUNEL staining and the quantitative results of heart tissues in each group (*n* = 6; scale bar, 50 μm). *P < 0.05 compared with the Saline group. ^#^*P* < 0.05 compared with the LPS+WT group.

Western blot results showed that LPS stimulation increased the protein expression of Bax and C-caspase3 and decreased the protein expression of Bcl-2 in hearts. These changes were obviously aggravated by IL-12p40 deletion (Figure 5A). Moreover, compared with the saline group, the number of TUNEL-positive cells in the hearts of the LPS group was significantly increased and was further increased by IL-12p40 deletion (Figure 5B).

### WT monocyte adoptive transfer alleviates cardiac injury in LPS-treated IL-12p40^–/-^ mice

**Figure 6:**
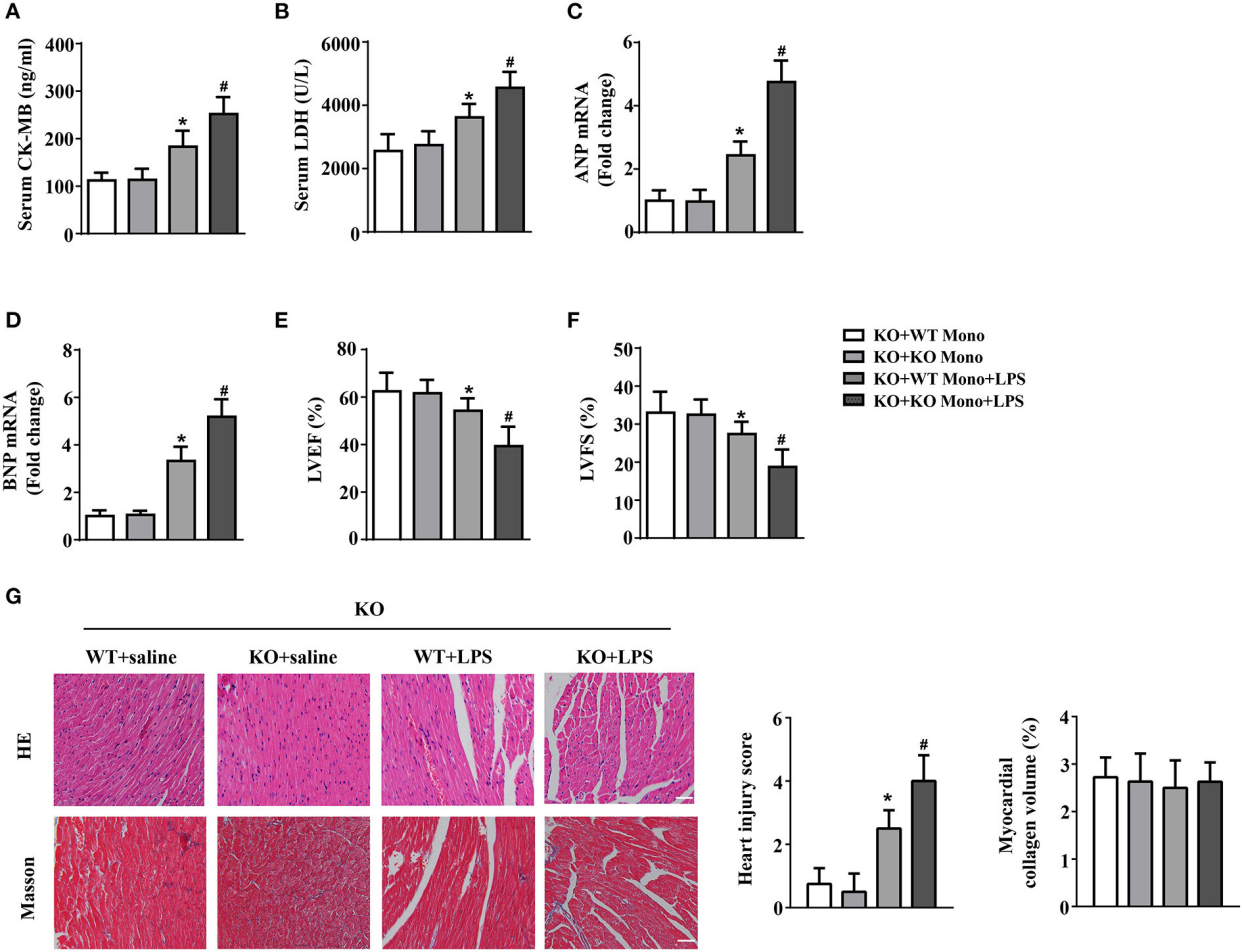
WT monocyte adoptive transfer alleviates cardiac injury in LPS-treated IL-12p40^−/−^ mice. **(A,B)** The levels of LDH and CK-MB in serum of mice in each group (*n* = 6). **(C,D)** qRT-PCR analysis of ANP and BNP mRNA expression levels in the hearts of mice in each group (*n* = 6). **(E,F)** Echocardiography analysis of LVEF and LVFS of mice in each group (*n* = 6). **(G)** HE and masson's trichrome stainings and the quantitative results of heart tissues in each group (*n* =6; scale bar, 100 μm). *P < 0.05 compared with the KO+WT Mono group. ^#^*P* < 0.05 compared with the KO+WT Mono+LPS group.

To explore the effects of exogenous monocytes on LPS-treated IL-12p40^−/−^ mice, WT or IL-12p40^−/−^ mouse monocytes were injected *via* the tail vein prior to LPS or saline treatment. Our results showed that lower levels of LDH and CK-MB in serum and lower mRNA levels of ANP and BNP in the hearts were observed in LPS-treated IL-12p40^−/−^ mice with adoptive transfer of WT monocytes than those in LPS-treated IL-12p40^−/−^ mice with adoptive transfer of IL-12p40^−/−^ monocytes (Figures 6A–D); however, these significant differences were not observed in saline-treated IL-12p40^−/−^ mice (Figures 6A–D). In addition, the echocardiography results also showed that adoptive transfer of WT monocytes could improve cardiac function of LPS-treated IL-12p40^−/−^ mice (Figures 6E,F). Moreover, the results of histological examination indicated that adoptive transfer of WT monocytes can decrease heart injury scores of LPS-treated IL-12p40^−/−^ mice but have no effect on the myocardial collagen volume (Figure 6G).

### WT monocyte adoptive transfer alleviates myocardial apoptosis in LPS-treated IL-12p40^–/–^ mice

**Figure 7:**
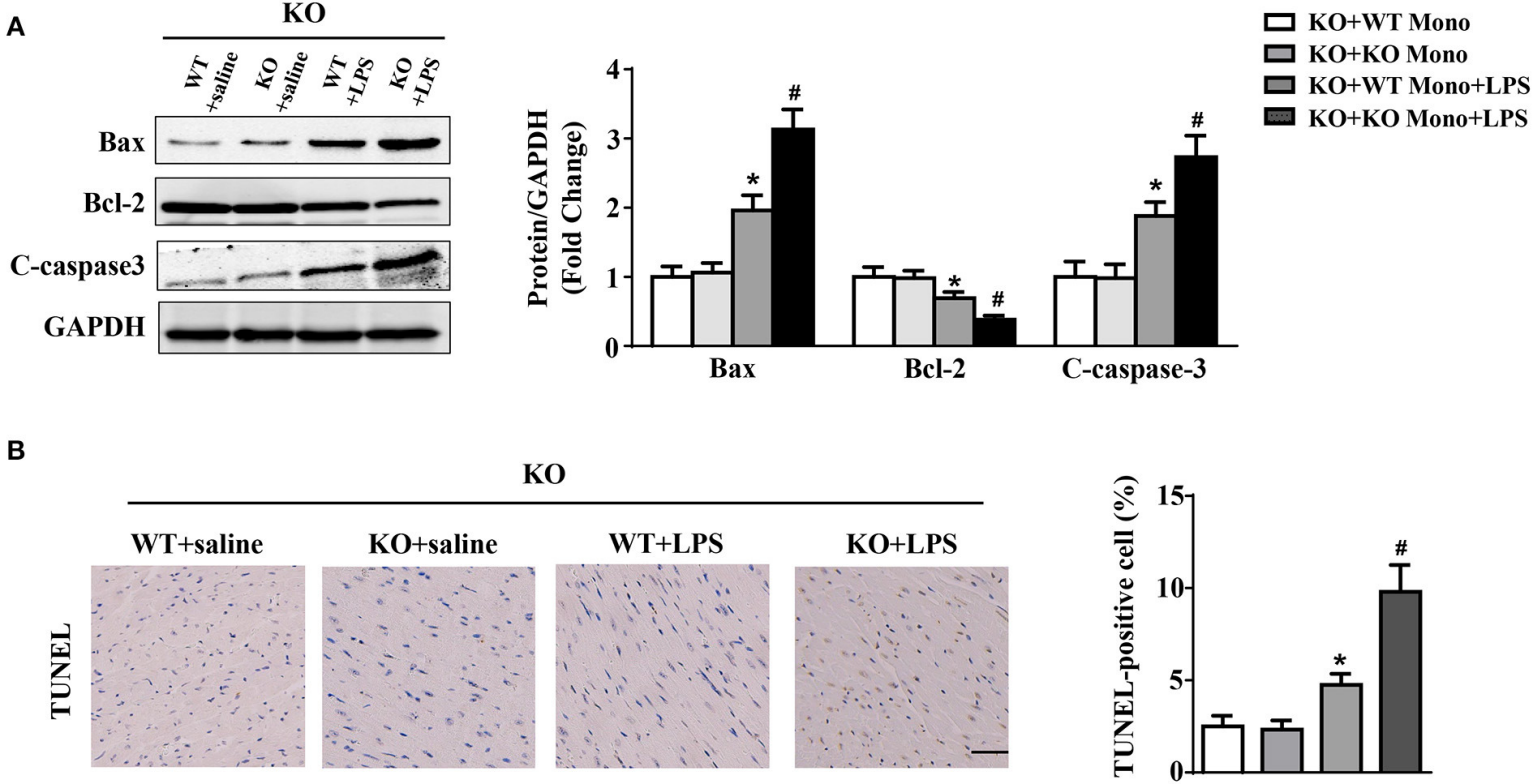
WT monocyte adoptive transfer alleviates myocardial apoptosis in LPS-treated IL-12p40^−/−^ mice. **(A)** Western blot analysis of Bax, Bcl-2 and C-caspase-3 protein levels in heart tissues of each group (*n* = 5). **(B)** TUNEL staining and the quantitative results of heart tissues in each group (*n* =6; scale bar, 50 μm). *P < 0.05 compared with the KO+WT Mono group. ^#^*P* < 0.05 compared with the KO+WT Mono+LPS group.

Adoptive transfer of WT monocytes into LPS-treated IL-12p40^−/−^ mice decreased the protein expression of Bax and C-caspase-3 and increased the protein expression of Bcl-2; however, these significant differences were not observed in saline-treated IL-12P40^−/−^ mice (Figure 7A). In addition, LPS-treated IL-12p40^−/−^ mice with adoptive transfer of WT monocytes exhibited fewer TUNEL-positive cells than those with adoptive transfer of IL-12p40^−/−^ monocytes (Figure 7B).

### WT monocyte adoptive transfer inhibits cardiac inflammation in LPS-treated IL-12p40^–/–^ mice

**Figure 8:**
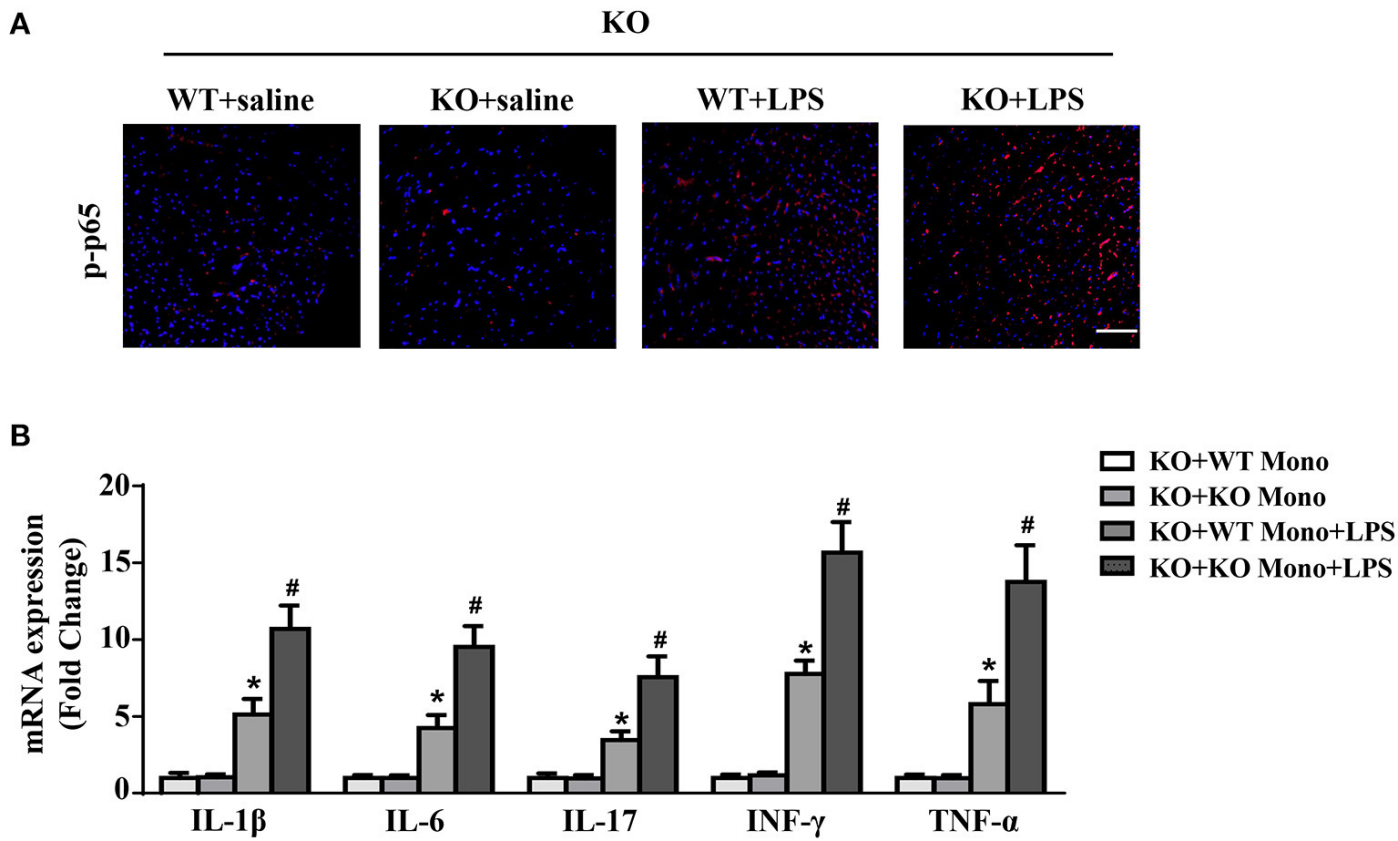
WT monocyte adoptive transfer reduces cardiac inflammation in LPS-treated IL-12p40^−/−^ mice. **(A)** The immunofluorescence analysis of p-p65 in heart sections of each group (*n* = 6; scale bar, 50 μm). **(B)** qRT-PCR analysis of IL-1β, IL-6, IL-17, TNF-α, and INF-γ mRNA expression levels in the hearts of mice in each group (*n* =6). *P < 0.05 compared with the KO+WT Mono group. ^#^*P* < 0.05 compared with the KO+WT Mono+LPS group.

Compared to the LPS-treated IL-12p40^−/−^ mice with adoptive transfer of IL-12p40^−/−^ monocytes, the levels of p-p65 were significant lower in the hearts of LPS-treated IL-12p40^−/−^ mice with adoptive transfer of WT monocytes (Figure 8A). Moreover, the inhibitory effect of WT monocyte adoptive transfer on inflammation was further confirmed by the results of qRT-PCR, which showed that the mRNA expression of proinflammatory cytokines, including IL-1β, IL-6, IL-17, TNF-α, and INF-γ were significantly down-regulated in LPS-treated IL-12p40^−/−^ mice with adoptive transfer of WT monocytes (Figure 8B).

## Discussion

In this study, we explored the effects of IL-12p40 deletion on LPS-induced cardiac dysfunction and elucidated the underlying mechanisms. We found that the expression of IL-12p40 was upregulated in mice after treatment with LPS. In addition, our findings indicated that IL-12p40 deletion aggravated cardiac injury and cardiac dysfunction and increased monocyte infiltration in mice treated with LPS. Moreover, IL-12p40 deletion enhanced the phosphorylation of NF-κB and MAPK signaling pathways, and up-regulated the expression of inflammatory factors. In subsequent experiments, we found that the levels of cardiac inflammation and cardiac injury in LPS-treated IL-12p40^−/−^ mice with adoptive transfer of WT monocytes were lower than those in LPS-treated IL-12p40^−/−^ mice with adoptive transfer of IL-12p40^−/−^ monocytes.

The results of epidemiological statistics showed that ~40% of patients with sepsis have cardiac dysfunction (34). This type of cardiac dysfunction is caused by sepsis alone and is called SIC. Previous studies have demonstrated that the mortality rate of septic patients with SIC is obviously higher than that of septic patients without SIC (1, 35). Although the underlying mechanisms of SIC have been explored by many studies, these processes are not completely understood. Accumulating evidence indicates that inflammatory reactions are key factors for the initiation and progression of SIC (6). Interleukins (ILs) play an important role in regulating the immune system of human beings. There are more than 40 ILs which can be divided into six families according to their biological functions. IL-12 and IL-23 are the two proinflammatory factors of the IL-12 family and share a common subunit of IL-12p40. Thus, knocking out or neutralizing the IL-12p40 subunit can counteract the biological effects of both IL-12 and IL-23. To date, there is growing evidence that neutralizing the IL-12p40 subunit can improve the prognosis of many patients with autoimmune diseases by regulating the immune/inflammatory response (36–38). In this study, we detected that IL-12p40 expression in the hearts of mice was upregulated within 6 h after treatment with LPS; however, after 12 h, the expression of IL-12p40 showed a downwards trend. The mechanism of this phenomenon is unclear and may be attributed to the inflammatory overreaction in the early stage of sepsis, followed by immunoparalysis or immunosuppression. Moreover, we also detected that IL-12p40 deletion upregulated the expression of inflammatory factors and aggravated cardiac injury and cardiac dysfunction in mice treated with LPS. Therefore, we speculated that IL-12p40 deletion could aggravate LPS-induced SIC in mice.

The NF-κB pathway is considered an important proinflammatory signaling pathway and can mediate the synthesis of cytokines, including TNF-α, IL-1β, IL-6, IL-8, etc. NF-κB is a heterodimer and p65 is one subunit of it. Sakurai et al. reported that IL-2 deletion leads to the development of inflammatory colitis accompanied by enhanced NF-κB activation (39). In addition, Wang et al. found that sevoflurane can ameliorate LPS-induced inflammatory injury of HK-2 cells by down-regulating the expression of p-p65 (40). MAPK signaling pathway is one of TLR4-related immune signalings and has three major subfamilies, including ERK, JNK and p38 (41). TLR4 is responsible for the recognition of LPS and MAPK signaling pathway is involved in regulating cardiac inflammatory responses triggered by LPS (28). STAT1 is an important mediator of biological responses induced by inflammatory activators (42), and the JAK-STAT signaling pathway is known as a mechanism involved in immune regulation (43). In our study, the results indicated that the phosphorylation of p65, p38, ERK, and JNK were significantly increased in mice treated with LPS, and IL-12p40 deletion further exacerbated these changes. However, neither LPS stimulation nor IL-12p40 deletion had significant effect on the phosphorylation of STAT1 in mice. Therefore, IL-12p40 deletion aggravates LPS-induced cardiac dysfunction in mice by activating NF-κB and MAPK signaling pathways but not the JAK-STAT1 signaling pathway.

Monocytes are natural immune cells that can participate in the activation of the innate immune system and release inflammatory cytokines and chemokines after recognized pathogens. The cytokines include TNF-α, IL-1β, IL-6, IL-12, IL-18, and IL-23 (44, 45), while the chemokines include CCL2/MCP-1, CXCL8, CXCL10, CCL18, and CCL20 (46–48). Then, these cytokines and chemokines further activate and recruit other immune cells to the inflammation sites and trigger a series of inflammatory responses (49). In recent years, many studies have reported the role of monocytes in sepsis. Gainaru et al. found that the circulating monocyte count was greatly increased in gram-negative sepsis (32). Raffray et al. observed that zoledronate could rescue immunosuppressed monocytes during acute sepsis and thus may help improve clinical outcomes during severe infection (50). Furthermore, Sáenz et al. reported that we could diagnose severe sepsis in the early stage by analyzing the monocyte immunophenotype (51). In this study, we found that the infiltration of monocytes in mice was significantly increased after treatment with LPS. Moreover, IL-12p40 deletion further increased the infiltration of monocytes in mice treated with LPS.

However, the role of monocytes in LPS-induced cardiac dysfunction in IL-12p40^−/−^ mice still needs to be further verified. In subsequent experiments, we transferred WT and IL-12p40^−/−^ monocytes into different IL-12p40^−/−^ mice treated with LPS. The results indicated that transfer of WT monocytes can significantly alleviate cardiac inflammation and cardiac injury compared with transfer of IL-12p40^−/−^ monocytes. One possible explanation is that WT monocytes can secrete more IL-12 and IL-23 than IL-12p40^−/−^ monocytes, and transfer of WT monocytes can partially offset the loss of the biological effects of IL-12 and IL-23 caused by IL-12p40 deletion. Thus, monocytes can regulate LPS-induced inflammatory responses, cardiac injury and cardiac dysfunction by secreting IL-12 and IL-23, and their functions are different in different environments.

In conclusion, our research indicated that IL-12p40 deletion significantly aggravated LPS-induced cardiac injury and cardiac dysfunction in mice by regulating the NF-κB and MAPK signaling pathways, and this process may be related to monocytes. Therefore, IL-12p40 show a protective role in SIC, and IL-12p40 deficiency or anti-IL-12p40 monoclonal antibodies may be detrimental to patients with SIC.

## Data availability statement

The original contributions presented in the study are included in the article/Supplementary material, further inquiries can be directed to the corresponding author/s.

## Ethics statement

The animal study was reviewed and approved by Animal Care and Use Committee of Renmin Hospital of Wuhan University.

## Author contributions

ML, ZW, and JZ contributed to the experimental design and wrote the manuscript. DY, MW, YX, MZ, JL, and JY contributed to the acquisition and analysis of the data. YF, XL, WP, HP, CW, DT, WL, and JW reviewed the manuscript. All authors contributed to the article and approved the submitted version.

## Funding

This work was supported by the National Natural Science Foundation of China (No. 1382070436 to JW).

## Conflict of interest

The authors declare that the research was conducted in the absence of any commercial or financial relationships that could be construed as a potential conflict of interest.

## Publisher's note

All claims expressed in this article are solely those of the authors and do not necessarily represent those of their affiliated organizations, or those of the publisher, the editors and the reviewers. Any product that may be evaluated in this article, or claim that may be made by its manufacturer, is not guaranteed or endorsed by the publisher.
