# Supplementary figures and images for: IL-12p40 deletion aggravates lipopolysaccharide-induced cardiac dysfunction in mice
Source: Front Cardiovasc Med. 2022 Sep 16;9:950029. doi: 10.3389/fcvm.2022.950029 (PMC9523082; doi:10.3389/fcvm.2022.950029)

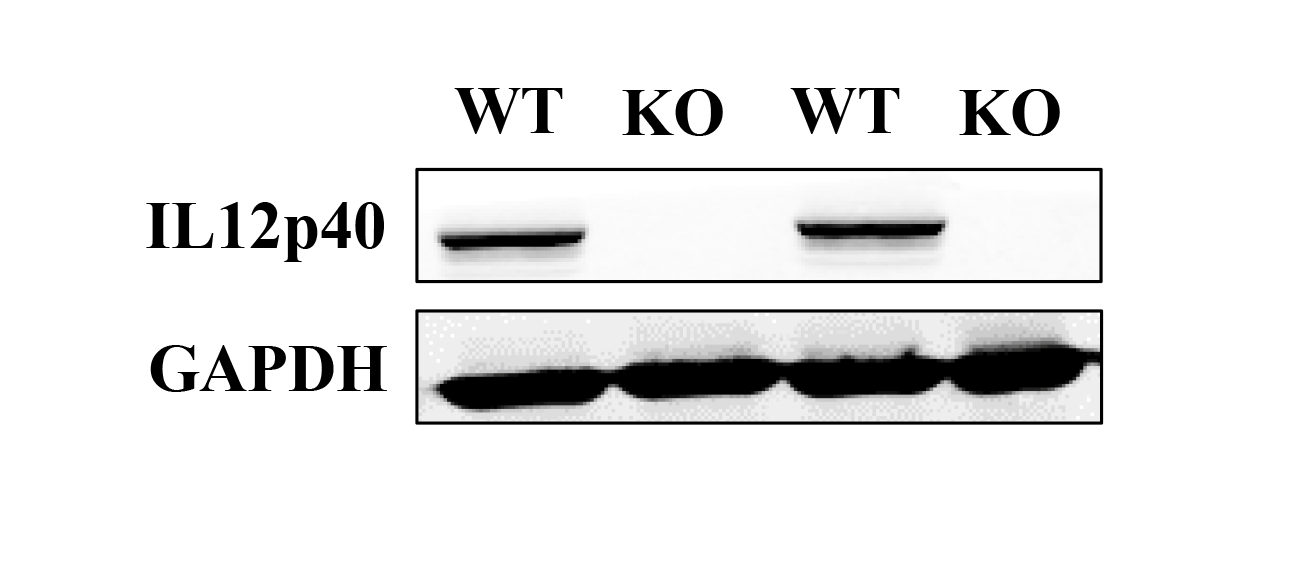

Supplement: Supplementary file 8 [file Image_1.TIF]
